# Detection Strategies for COM, WMI, and ALPC-Based Multi-Process Malware

**DOI:** 10.3390/s24165118

**Published:** 2024-08-07

**Authors:** Radu Marian Portase, Andrei Marius Muntea, Andrei Mermeze, Adrian Colesa, Gheorghe Sebestyen

**Affiliations:** 1 Computer Science Department, Technical University of Cluj Napoca, 400114 Cluj Napoca, Romania; 2 Bitdefender, 060071 Bucharest, Romania

**Keywords:** malware, sensor evasion, behavior detection, COM, WMI

## Abstract

Behavioral malware detection is based on attributing malicious actions to processes. Malicious processes may try to hide by changing the behavior of other benign processes to achieve their goals. We showcase how Component Object Model (COM) and Windows Management Instrumentation (WMI) can be used to create such spoofing attacks. We discuss the internals of COM and WMI and Asynchronous Local Procedure Call (ALPC). We present multiple functional monitoring techniques to identify the spoofing and discuss the strong and weak points of each technique. We create a robust process monitoring system that can correctly identify the source of malicious actions spoofed via COM, WMI and ALPC with a low performance impact. Finally, we discuss how malicious actors use COM, WMI and ALPC by examining real-world malware detected by our monitoring system.

## 1. Introduction

Malware poses a significant threat to companies due to its ability to compromise sensitive data, disrupt operations, and inflict financial damage. Cybercriminals use malware to steal confidential information, hold systems hostage through ransomware, and cause widespread operational disruptions. The increasing sophistication of malware attacks makes it difficult for organizations to detect and respond to threats promptly. As a result, businesses face potential losses from data breaches, legal liabilities, and reputational damage, underscoring the importance of robust malware detection and prevention strategies.

To combat these threats, organizations establish dedicated units known as Security Operation Centers (SOCs) to monitor and manage security-related events across their IT infrastructure [1]. An SOC oversees various IT assets, including networks, firewalls, endpoint protection solutions, application servers, databases, and more. These events are typically generated by a diverse array of sensors and technologies. Analysts use a Security Incident and Event Management System (SIEM) to collect, normalize, and analyze all logs [2].

One critical component connected to an SIEM is the Endpoint Detection and Response (EDR) technology [3]. EDR encompasses tools and technologies focused on detecting and investigating suspicious activities on endpoints. It provides malicious activity detection, security-relevant event generation, storage, and retrieval.

EDRs need to monitor application behavior. Application behavior can be defined as a series of actions that have lasting effects on the operating system (OS) [4]. Examples include changes to the file system, modifications to the Windows registry, code injection into other processes, or the registration, and the starting and stopping of Windows services and tasks.

EDR requires sensors to be installed on machines within the infrastructure. These sensors can be bypassed by leveraging operating system features. Microsoft Windows, one of the oldest and most widely used operating systems globally, is prevalent on both personal computers and enterprise workstations and servers. Due to the age of the OS, it has many legacy technologies that can help malware bypass EDR solutions.

In this paper, we analyze *spoofing attacks*, where malware executes actions out-of-process or across multiple processes to evade dynamic malware detection systems. We examine how COM and Windows Management Instrumentation (WMI) can be used for such attacks due to their inherent out-of-process execution models. We also delve into how COM is built over Asynchronous Local Procedure Call (ALPC) and demonstrate how malware can leverage ALPC for defense evasion.

COM [5] is a Microsoft standard developed in 1993 for creating software components. It enables programs to interact with components written for other programs. For example, Microsoft Outlook can display documents or tables using Microsoft Word and Excel rendering engines without duplicating their functionality. COM components can be loaded as dynamic link libraries or, more interestingly from an anti-malware evasion perspective, reside in other server processes. In the latter case, the server executes any action requested by the client, i.e., the malware, thereby concealing the malware’s actions behind the server.

WMI [6] is the infrastructure for management data and operations on Windows-based operating systems. WMI can perform many actions deemed dangerous from a dynamic behavioral detection perspective. For instance, WMI can obtain system information, interact with the Windows registry, create processes, or achieve post-reboot persistence [7]. Interacting with WMI at the binary level is accomplished through COM, which directly translates to out-of-process execution and, as we demonstrate later, defense evasion.

Asynchronous Local Procedure Calls (ALPC) is an internal mechanism within the Windows operating system designed for inter-process communication (IPC) on the same machine [8]. Although Microsoft has not extensively documented ALPC, it is fundamentally integrated within the Windows ecosystem, notably in interactions involving COM components. A critical function of ALPC is its role in facilitating Windows Remote Procedure Calls (RPCs).

RPCs in Windows enable distributed computing by abstracting the complexities associated with network communications. This capability allows for resource sharing, enhances modularity and scalability, ensures location transparency, and promotes interoperability. Additionally, RPC optimizes performance, improves fault tolerance and reliability, and supports centralized management and security.

RPC accommodates multiple data transfer protocols. For instance, it can utilize TCP/IP, employ named pipes, or opt for Local Inter-process Communication. The latter leverages the ALPC mechanism for data exchanges. This latter case is also the one that is relevant for the spoofing attacks we study in this paper. A process may connect to another process’s RPC port and request actions to be performed on its behalf.

COM and WMI have become critical attack vectors against malware detection systems in recent years. At DEF CON 2017, Sean Dillon and Zach Harding presented Koadic [9], which is a post-exploitation framework and remote access tool entirely based on COM and WMI. The idea of using COM and WMI for evasion is not new. Empire [10], a post-exploitation framework written for PowerShell and released by Will Schroeder and Justin Warner in 2015, also uses the *Win32_Process* [11] WMI class [12] to create processes. Even the infamous *Vault 7: CIA Hacking Tools Revealed* leak from WikiLeaks [13] contains tutorials on using WMI for stealthy information gathering. In addition, early advanced persistent threats like Stuxnet [14] utilized WMI in some forms.

Attackers can leverage various RPC interfaces for purposes like the enumeration of users, groups of users or domain information; some interfaces even allow the creation or deletion of users in a domain [15] or lateral movement [16].

Despite the popularity of COM, WMI and RPC for defense evasion, we believe that defending against these technologies as methods for action source spoofing attacks is insufficiently discussed.

The main contributions of this paper are outlined below:

- We formulate the problem introduced by the out-of-process execution nature of COM to dynamic detection.
- We critically discuss how a malware detection system can monitor COM, WMI, and RPC over ALPC activity using various degrees of intrusion, starting with the classic, very intrusive, binary interception methods and finishing with two new, non-intrusive techniques created by us based on observing undocumented Windows functionality.
- We design and implement a dynamic malware detection solution capable of intercepting COM, WMI, and ALPC activity with a low performance impact.
- We use our monitoring system to measure the spread of evasive malware that uses COM, WMI, and ALPC directly for action spoofing in a large dataset.

The paper is structured as follows. In Section 2, we present related work on anti-malware evasion with a focus on how dynamic detection engines can be bypassed via spoofing attacks. Section 3 showcases how COM and WMI can be used to spoof the source of malicious action. In Section 4, we present some details about how COM works. In Section 5, we analyze several strategies to monitor COM object usage. In Section 6, we discuss how ALPC further complicates the issue of detecting spofed actions. In Section 7, we discuss a possible behavioral detection architecture and show how the COM monitoring strategies can be used to improve the detection rate and system disinfection. In Section 8, we discuss detection improvement and the performance impact introduced by monitoring COM. In Section 9, we discuss our methodology and findings and address some aspects about protection against COM, WMI and ALPC malware. We conclude this paper with Section 10.

## 2. Related Work

Malicious action source spoofing attacks are recognized and studied by the security research community. In [17], Ji, He, Zhu, Li, and Guo propose a multi-process mechanism for evading behavior-based bot detection by separating the command and control (C&C) connection from malicious behaviors and distributing malicious activities across multiple processes. This method significantly reduces the effectiveness of conventional detection techniques that monitor single-process behaviors. Experiments with a multi-process bot, derived from a simplified Zeus bot, show a marked decrease in detection probability compared to a single-process bot. In [18], the authors create malWASH to study the effect of injecting parts of the payload into clean processes running on a system. Their proposed approach effectively hides the malware’s behavior within benign processes with negligible performance impact, making detection by behavioral analysis tools significantly more difficult. Even if some processes are terminated, malWASH ensures continuous execution by transferring control to the remaining processes. Ma et al. [19] even developed a prototype tool that can translate any malware code to multiple “shadow” processes. Each “shadow” process executes a part of the payload so that the complete behavior of the original malware is maintained.

All previously mentioned works rely on code injection for multi-process execution that can be detected by security solutions. We showcase a different approach to split malicious behavior in multiple processes by simply relying on COM, WMI and RPC as replacements for in-process calls to Windows APIs. Our approach is more stealthy because we use the existing functionality of the operating system.

COM is a very old technology. Books and articles about it were mostly published before the year 2000. In [20], the authors discuss the internals of Distributed COM and show some practical examples on how to use it. The authors of [21] give more details about the internals of COM and how it can be used in applications. A more recent presentation of COM is [22]. Our article extracts a short summary of COM internals from this existing work, and we focus on how COM can be used for defense evasion.

Some existing works discusses how COM objects can be hijacked for persistence [23]. Our work is about how malware can use COM as it was designed for defense evasion. We ignore COM hijacking entirely.

As mentioned in the introduction, post-exploitation frameworks like Koadic [9] were created specifically to showcase how COM can be used for defense evasion. We test how many malware have adopted COM and WMI for this purposes by using a large collection of malware.

Ideas for monitoring COM have existed for a long time (Keith Brown’s Universal Delegator [24,25] was presented in 1999). In our paper, we show why the Universal Delegator cannot be used to monitor WMI and provide multiple less intrusive alternatives based on modern technologies like Event Tracing for Windows.

WMI is recognized as one of the techniques used by the so-called fileless malware [26]. In 2015, Matt Graeber’s presentation at Black Hat [27] even showed how one can build a fully functioning backdoor using it. Existing work on defending against WMI malware like [28,29] focus on how WMI is used for persistence and how it can be detected on disk after a compromise. We focus on real-time detection and provide solutions on how to monitor WMI usage on modern Windows Operating Systems.

RPC monitoring is becoming popular. Techniques for RPC monitoring sometimes are related to COM, WMI and ALPC monitoring.

Patent [30] shows how RPC can be monitored using proxy server objects. Setting the proxy can be accomplished using API hooking. We also use hooks to monitor ALPC. Our solution requires less knowledge about the internals of the protocols.

Patent [31] describes a monitoring method based on receiving notifications of kernel-mode events linked to the user-mode processing of request messages by RPC-utilizing processes. By utilizing data structures like the Thread Environment Block (TEB) and specific RPC-related fields within the operating system, the agent traces and retrieves the request message. This allows for the identification of the originator of the request. We similarly use the inspection of fields from the TEB for monitoring COM object usage. We integrate this technique in a more complex multi-layered monitoring system for COM, WMI and ALPC.

In their 2023 patent [32], Gen Digital suggests a novel method that connects the Background Intelligent Transfer Service (BITS) with the source program that initiates it. This method intercepts file operations, like creating or renaming files, carried out by an instance of svchost.exe—the service responsible for managing BITS tasks. The key concept involves verifying if the thread performing these operations is associated with any ALPC messages and examining the content of these messages at the Kernel level. However, a significant limitation of this approach is its inapplicability to all types of actions, since it necessitates a transition to Kernel space, which is usually through a system call. By that stage, the ALPC message might have already been destroyed or invalidated. Interacting with BITS can be accomplished with various COM objects [33]. Our paper looks at how to monitor COM and ALPC more generically; this includes monitoring of BITS protocol.

Hajmasan et al. [34] created a solution for detecting multi-process malware by changing the classic definition of process behavior to group behavior. Process groups are created and managed by an entity manager that analyzes process creations and code injections. The proposed solution uses various sensors that send information to an internal heuristic engine for malware detection. The heuristics assign a score to either a group or a process contained in it. Detection is triggered when a certain threshold for group or process score is reached. After detection, a remediation module is notified about malicious actions performed by a group and attempts to revert those actions. Our paper shows how this solution may be insufficient when dealing with COM, WMI and RPC via ALPC. We propose and build an extension of Hajmasan’s system that integrates COM, WMI and ALPC monitoring capabilities with the entity manager and the heuristic engine. Our improved system can detect more malware with a low performance impact.

## 3. Problem Statement: Effects of Out-of-Process Execution on Behavioral Detection

Windows provides many technologies that can be used together to monitor process behavior. Usually, security solutions use minifilter [35] drivers to monitor file system activity, Kernel Callback Functions to monitor process and thread creation, module loads, and registry activity [36], and the Windows Filtering Platform [37] to monitor network activity.

Microsoft’s latest work on application monitoring revolves around Event Tracing for Windows (ETW) [38]. This technology allows application programmers to start and stop event tracing sessions, instrument an application to provide trace events, and consume trace events. Most Windows components are instrumented to use ETW for logging and debugging purposes. The latest versions of Windows 10 include the *Microsoft-Windows-Threat-Intelligence* (GUID: *4e1897c-bb5d-5668-f1d8-040f4d8dd344*) provider, which notifies any active listeners on function calls related to code injection, such as allocating, writing or changing protection for memory located in another process.

Direct library call interception, also known as *function hooking*, is used by security solutions when no other technological alternative for monitoring an action exists. Microsoft has a neutral stance about hooking. The monitoring technique is not directly supported, but Microsoft sometimes uses it for protection [39] or application compatibility shims [40].

Knowing how applications running on Windows are monitored, we created a simple experiment to check what would be seen by an application behavior monitor if we replaced direct Windows API calls with COM (and WMI through COM) alternatives. We provide detailed code for the COM versions so that the reader can notice that the COM code is not much more complicated than simply calling the APIs. The development time for the two examples with direct calls to the Windows API and the COM version are similar.

For our experiment we propose and use the following simple flow:

1. Copy *powershell.exe* in the current directory;
2. Register the copy to run at each boot using the known
  *HKCU\Software\Microsoft\Windows\CurrentVersion\Run* persistence registry key;
3. Finally, start the copy of PowerShell with a harmless command line which displays a string in the console.

Firstly, we created a non-evasive sample by chaining the Win32 API functions *CopyFileW*, *RegSetValueEx* and *CreateProcess*. We named this non-evasive sample *NonComExamples.exe* to differentiate from the evasive COM-based implementation. Secondly, we created an evasive sample based on COM by replacing the APIs with their respective COM counterparts.

For *CopyFileW*, we used *IFileOperation* [41] using a code which can be simplified to the following:


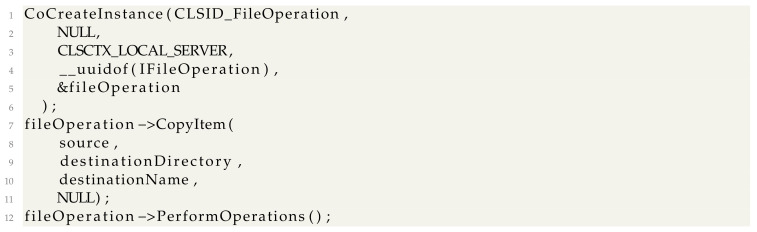


For registry and process creation operations, we used WMI operations through the standard COM interface [42]. The logic is simple; we obtain the initial locator to Windows Management on this computer, connect to the *ROOT\\CIMV2* WMI namespace with the current user, and obtain the pointer to the *IWbemServices* COM object. Using *IWbemServices*, one can access any WMI class and call methods on them. We replace *RegSetValueEx* and *CreateProcess* with calls to the WMI classes *StdRegProv* [43] and *WIN32_Process*. Code is implemented as follows:


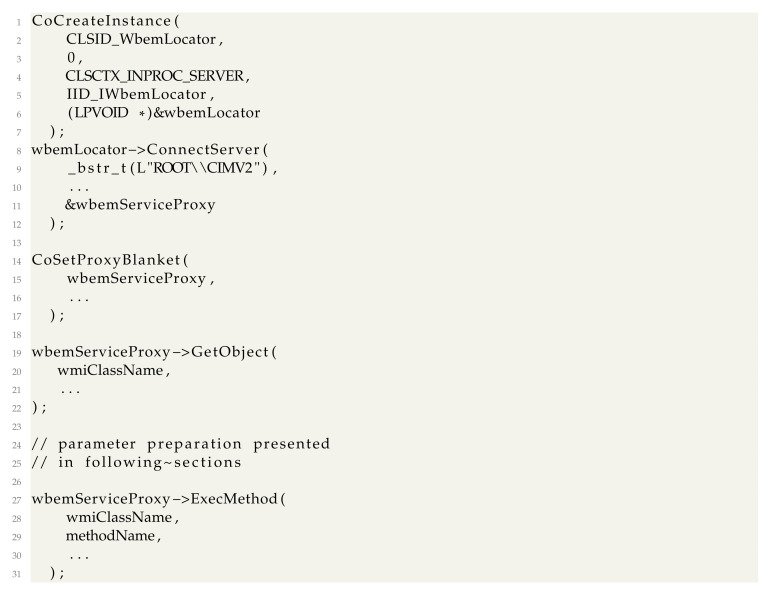


**Figure 1:**
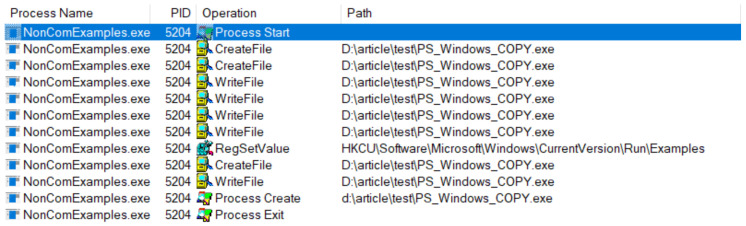
Procmon result for in-process execution using Win32 Api.

**Figure 2:**
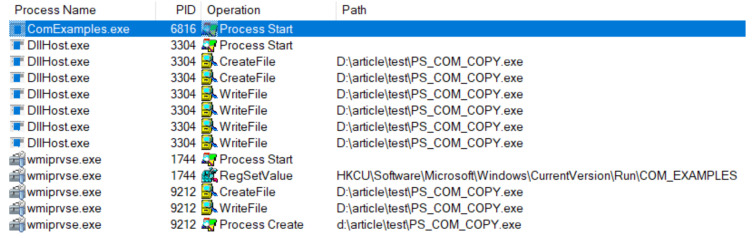
Procmon result for out-of-process execution using COM.

We use the well-known Sysinternals Procmon [44] as the application monitoring software for our experiment because it uses a minifilter driver and kernel notification routines similar to most anti-malware solutions. Procmon can see the actions of our basic malware when they are executed using Win32 API, as illustrated in Figure 1. For our COM implementation, the file copy operation is performed by a *dllhost.exe*. The Registry changes and the process creations are performed by two *wmiprvse.exe* processes, as illustrated in Figure 2. We explain why this happens later in the paper, but for the moment, we only note that the source (i.e., the process) of the recorded operations is hidden behind legitimate system processes. This essentially causes the spoofing attack; a security solution will see the system processes perform the operations, not the malware.

Our experiment can be replicated on Windows from Windows 7 up to the latest version of Windows 11 with the same result.

## 4. Understanding the Problem: COM and WMI Internals

In an effort to create a monitoring system capable of intercepting COM and WMI, we tried to understand how the technology works internally. We found some sources describing COM [21,22,45] and supplemented the missing or outdated information using various reverse engineering techniques. The figures presented in this section are based on the style used in [22].

Please note that we kept only information relevant to building our security solution in this section. COM has many more features that can be explored from an attacker or defender’s perspective.

### 4.1. COM Object Anatomy

**Figure 3:**
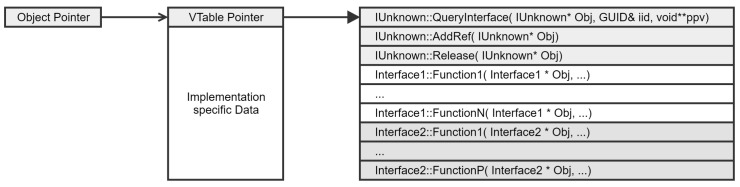
COM object anatomy.

COM objects are implemented in a similar manner to that of Visual C++ objects, as illustrated in Figure 3. The first field of each object is a pointer to a virtual function table (VTable) containing methods specific to the object. All methods in the VTable have a pointer to the object as a first parameter. Any other parameters follow.

All COM interfaces derive from the *IUnknown* [46] interface. This interface defines the identity of the object. *IUnknown* has two basic responsibilities: reference counting for object life cycle management and exposing the *QueryInterface* method for resolving object pointer casting problems.

COM objects may provide multiple interfaces. When multiple inheritance is used to implement several interfaces in one object, the compiler concatenates the virtual functions tables of the various interfaces. The order of inheritance decides the order in which their entries appear in the object’s memory layout.

Each COM interface has a unique id (IID), which is represented as a GUID [47] structure. The *QueryInterface* routine queries a COM object for a pointer to one of its interfaces, identifying the interface by a reference to its *IID*.

### 4.2. Creating a COM Object

All registered COM objects that can be created are listed under *HKEY_CLASSES_ROOT\CLSID\* as configuration subkeys named using the string form of their GUID unique identifier. Using the registry information, a developer can obtain a class factory [48] COM object which can be used to create other COM objects by calling the *CreateInstance* method.

The implementation details of how the objects are created are simplified in two functions, *CoGetClassObject* [49] and *CoCreateInstanceEx* [50]. This API allows programmers to create COM objects that run in the same process as the code instantiating the object, in another process on the same machine, or even in other machines.

### 4.3. COM Object Implementations

The container for the COM object implementations are called *COM servers* [51]. The simplest COM server is the In-Process Server. The registry configuration entries for this server contain the path of a dynamic link library (DLL) in the *InProcServer32* subkey. The implementation DLL is loaded into the process, and the class factory is created by calling the exported method *DllGetClassObject* [52].

**Figure 4:**
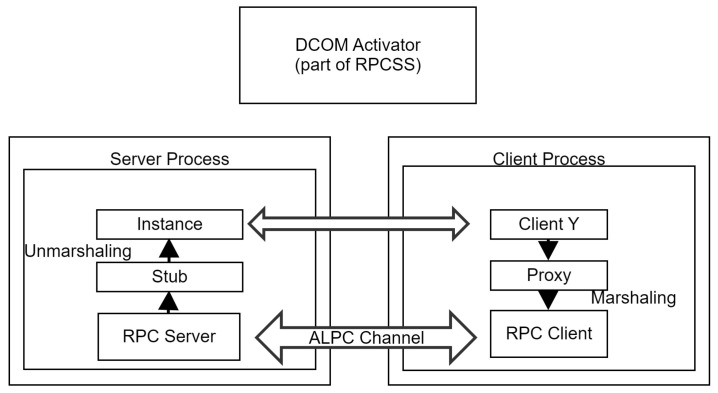
Local out-of-process server.

The Local Out-of-Process Server allows applications to use COM objects instantiated inside other processes on the same machine, as illustrated in Figure 4. The multi-process nature of this server implementation is hidden from the calling process through the use of a proxy [53] object (marshaler) on the client process, and a Stub [54] object (unmarshaler) on the server process. Communication is accomplished through the use of a named ALPC Channel [55].

The last kind of server allowed in COM is the Remote Out-of-Process Server also known as Distributed COM (DCOM). DCOM is a simple extension of the Local Out-of-Process server infrastructure, replacing ALPC with a TCP/IP channel.

### 4.4. Out-of-Process COM

**Figure 5:**
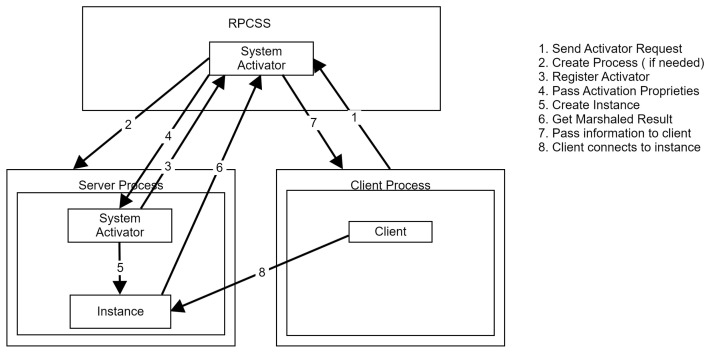
RPCSS system activator flow.

The creation of out-of-process COM objects can be accomplished either manually (by calling *CreateProcess*) or by simply specifying *CLSCTX _INPROC_SERVER* as parameter for *CoCreateInstance*. In the second case, COM will use internal components of the Remote Procedure Call (RPC) [55] standard implemented by Windows named the *System Activator* to obtain the object. The COM client process will ask the RPC server service (RPCSS) for an instance of the desired out-of-process object; RPCSS then locates or creates the server process and establishes communication (Figure 5). The flow is as follows:

1. Client sends activator request to the RPCSS system activator.
2. The activator creates or locates the server process.
3. System activator component of the server process registers with the RPCSS system activator.
4. RPCSS passes activation proprieties to the server.
5. Server creates an object instance.
6. RPCSS gets the marshaled result from the server.
7. RPCSS passes marshaled information to the client.
8. Client connects to out-of-process object instance.

Data between the client and the server are transmitted using Object Reference binary structures. Two types of object references exist: a standard reference (marshal by reference) which allows the calling of methods of a COM object located in a remote server and a custom reference (marshal by value) which resumes to instantiating COM objects inside the client process.

**Figure 6:**
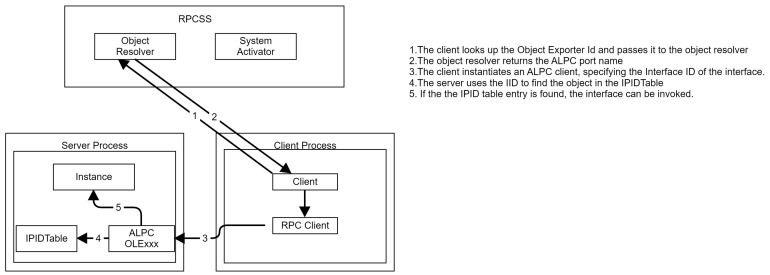
RPCSS object resolver flow.

The standard object reference creation flow (Figure 6) involved in out-of-process COM usage uses the Object Resolver component of RPCSS and is as follows:

1. The client looks up the object exporter ID and passes it to the RPCSS object resolver.
2. The RPCSS object resolver returns the ALPC port name corresponding to the server.
3. The client instantiates an ALPC client, specifying the Interface ID of the interface.
4. The server uses the Interface ID to find the object inside the server IPIDTable.
5. If the the IPID table entry is found, the interface can be invoked and the client can use the out-of-process COM object.

COM objects that can be instantiated out-of-process can be constructed as standalone executables, DLLs, or scripts. DLL components are typically executed inside a *dllhost.exe* process. This was seen in our example from Section 3. Components created as scripts, named scriptlets in COM terminology, are stored in an XML file and executed by the scriptlet run-time component *scrobj.dll*.

### 4.5. WMI as Seen from a COM Monitoring Perspective

There are several documented uses for WMI for defense evasion in malware. The first main one is using methods in WMI classes to interact with the system. In a previous section, we showed how any WMI class could be accessed through the *IWbemServices* COM object. A complete call to a class requires the generation of parameter objects which are passed to *IWbemServices*. This is accomplished using the *GetMethod* function of *IWbemClassObject* to obtain an input parameters definition object, then populating the input parameters object fields using the Put method and finally calling *IWbemServices::ExecMethod*.

A complete example of creating a process using the WMI class *WIN32_Process* and checking the result (after already having instantiated *wbemServiceProxy* as described in Section 3) can be resumed to the following: 


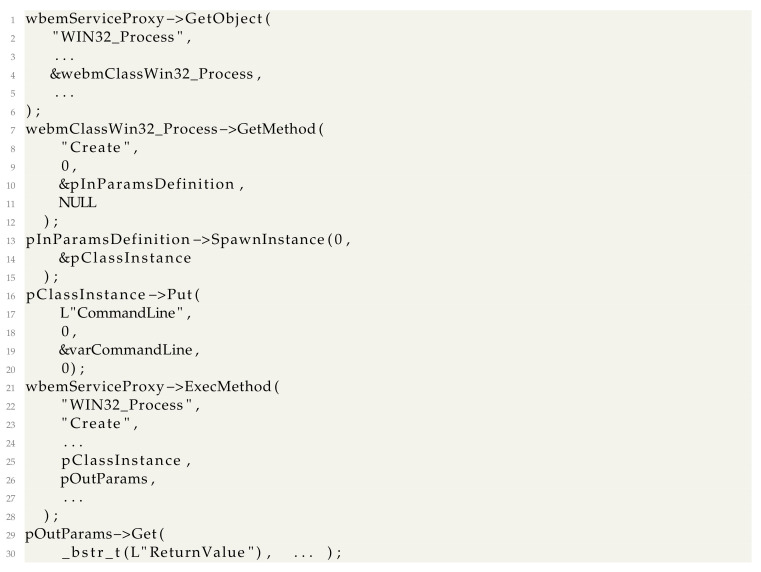


The second use for WMI is to use WMI specifiec queries (WQ) to interact with the operating system. In this case, the *IWbemServices::ExecQuery* method needs to be used. The query returns a COM object implementing the *IEnumWbemClassObject* interface. An example code flow could be:


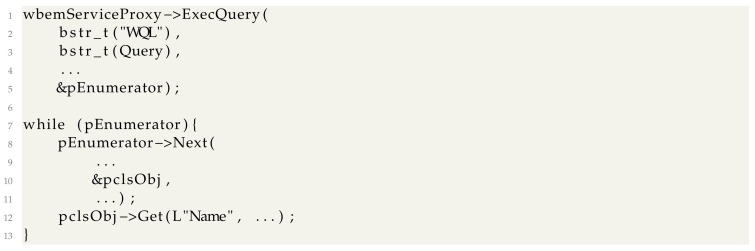


The third use for WMI is to register persistence through the event subscription feature. For this, a sample needs to create instances of a *CommandLineEventConsumer* or *ActiveScriptEventConsumer*, *__EventFilter* and *__FilterToConsumerBinding*. The instances are obtained using calls towbemServiceProxy->GetObject. After the instances are configured, they just need to be added to the WMI repositories using wbemServiceProxy->PutInstance. The complete process is described in [56].

Finally, some malware samples simply store payloads inside WMI repositories. They are out of scope for our solution, since this does not spoof the origin of any action.

Presenting the structure of the WMI queries, classes, and repository layouts are beyond this paper’s purpose. An excellent introduction to WMI as used by malware can be consulted in [7].

The *wmiprvse.exe* process seen in Section 3 is the WMI Provider Host. Providers are typically DLLs hosting COM objects, which extend the functionality of WMI.

When an application uses COM to interact with WMI, the client’s call goes to a *svchost.exe* process that hosts the “Windows Management Instrumentation Service” (winmgmt). The call is then forwarded from a worker thread to the wmiprvse.exe process that completes the request. In the case of our *ComExamples.exe* sample from Section 3, the first registry-related COM call leads to *svchost.exe*, which makes another COM call to a *wmiprvse.exe* process. This important aspect will affect several monitoring strategies discussed in the following section.

## 5. Building a Solution: Strategies for Monitoring COM and WMI Usage

Our main goal for monitoring COM and WMI is to correctly attribute the malicious actions to originating processes in order to be able to provide a complete list of actions for remediation in case malware is detected. An acceptable solution should run on any supported Windows version, starting with Windows 7, on both *x86* and *amd64* architectures.

We studied several ways to monitor COM activity by intercepting activity in the client, in the server, or by using already existing instrumentation data.

Acceptance testing for each monitoring strategy was accomplished by first checking if the implementation detects a series of synthetic tests extracted from real malware and post-exploitation framework behavior and performing stability tests for the solution using Microsoft Office and 5000 other randomly selected popular clean (i.e., malware-free) applications. A solution is considered viable only if Microsoft Office and the other clean applications install, start and stop correctly without crashing. In the following paragraphs, we present only viable monitoring strategies.

### 5.1. Client Monitoring: Object Proxies

**Figure 7:**
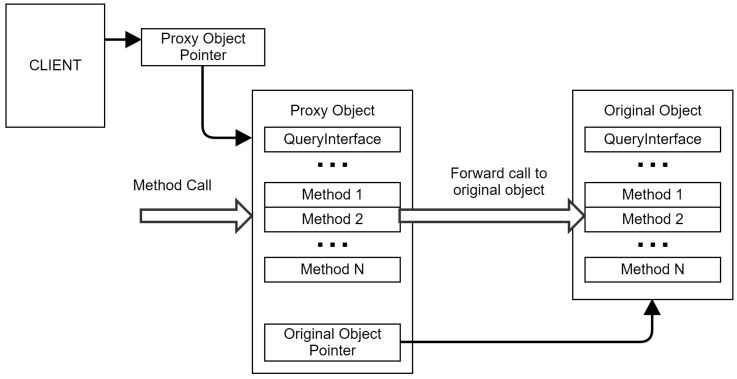
Monitoring COM through proxy objects.

COM has a built-in mechanism that provides a way to redirect one CLSID to another CLSID. Some articles [53] call this ability “emulation”, as one class can emulate another. According to the documentation, one should be able to replace a created instance of any COM object with a custom proxy object which has an interface identical to the source object, as illustrated in Figure 7. In the proxy, one could intercept any interesting calls to methods and forward the execution to the original object.

Setting up the proxy can be accomplished using the *CoTreatAsClass* [57] function, which sets a *TreatAs* registry subkey inside the COM objects registry configuration key.

We were able to avoid hooking any functions inside the client process by using a kernel-mode driver to virtualize the *TreatAs* registry key.

Keith Brown’s Universal Delegator [24,25] technique is frequently referred to in articles related to monitoring and intercepting COM usage. This delegator implements COM Object proxying that allows the arbitrary preprocessing and postprocessing of every method call of the proxied object when combined with a hook.

We have implemented a simplified *UniveralDelegator*, consisting of the *InterfaceDelegator* object, which wraps each interface that we want to monitor, and a *UniveralDelegator* class that manages these wrappers.

During our research, we realized that even though COM proxy objects work for simple objects, this solution does not work well for monitoring WMI-related *IWbemServices* objects. The major fault is in the *CoSetProxyBlanket* API we briefly showed in the code examples from Section 3. The API roughly translates to


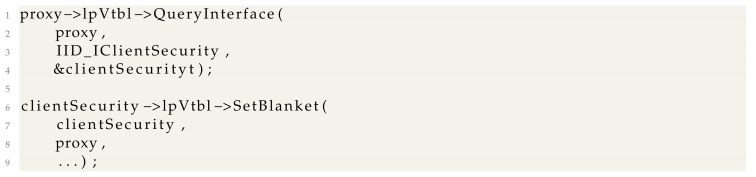


Decompiling *CClientSecurity::SetBlanket*, we see that it calls two functions: 


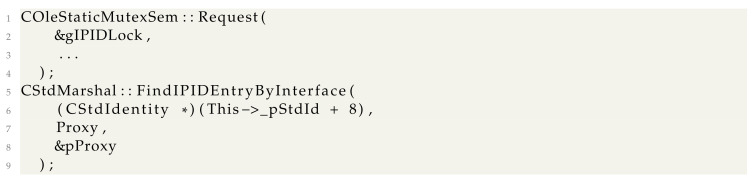


The *CStdMarshal::FindIPIDEntryByInterface* function walks a list of tagIPIDEntry* structures until the *IWbemServices* proxy for which the blanket should be set is found. If the *IWbemServices* proxy is not found, an error status is returned.


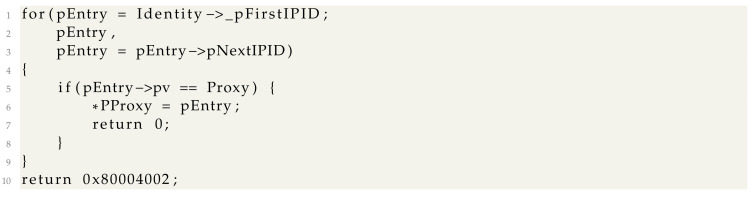


Because we set up a monitoring proxy, the second parameter of clientSecurity->lpVtbl->SetBlanket call is a COM interface that is wrapped in an *InterfaceDelegator*. This causes the (pEntry->pv == Proxy) check to always be false and *SetBlanket* to always fail.

We conclude that object proxies can be used to monitor non-WMI-related COM interfaces used by malware on all OS.

The proxy monitoring method has some degree of intrusion because a monitored application is forced to load a proxy object instead of the original object it requested.

The monitoring system can be bypassed if the malware sample decides to directly load required DLLs from the disk and instantiate COM objects from them without causing the COM framework to use the Registry.

### 5.2. Client Monitoring: Binary Call Interception

A binary call interception on functions and virtual function tables are known ways to monitor behavior. This kind of solution is similar to the object proxies mentioned before and can be used as a more generic way to monitor COM objects. As shown in Section 4, methods defined in a COM object can be called using an instance of the object in a similar manner to call methods inside C++ objects. In C., this would translate to something similar to the following:


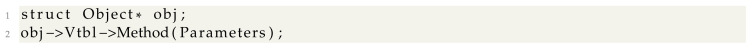


The caller does not care about the server used. If an out-of-process server is instantiated, the caller obtains a reference to a proxy which will forward all requests to the server through an identical interface to that obtained if the object would be instantiated inside the process.

**Figure 8:**
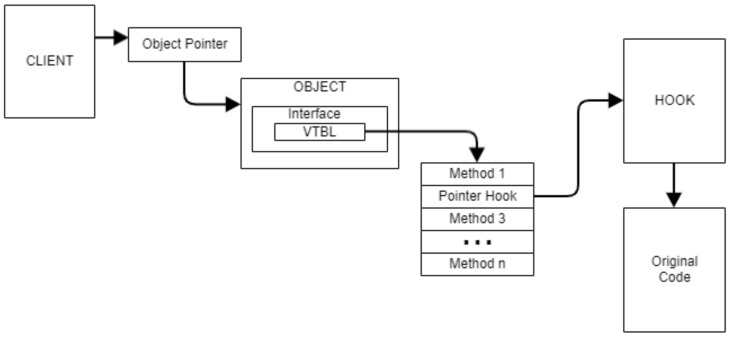
Monitoring COM through VTable patching.

Microsoft does not support hooks on member functions of COM objects. The functions are small and do not contain the mov edx, edx style instructions placed at the start of most exported library functions to support hot patching. We considered inline hooks at the start of the methods to be too dangerous to implement, so the only reasonable monitoring option left was VTable patching, as illustrated in Figure 8.

For implementing VTable patching, we used standard API hooking techniques to intercept calls to *CoCreateInstance* and *CoGetClassObject*. When a new object is created, we use *IUnknown::QueryInterface* for all interfaces we want to intercept, which were identified by their respective CLSIDs. We do this because a malicious sample might create an object with an interface we are not particularly interested in; then, we use *QueryInterface* to switch the interface, thus bypassing all monitoring. For this solution, we monitor WMI by intercepting calls to *IWbemServices* COM objects.

The binary call interception method works on all tested operating systems and provides reliable monitoring results. This solution has the main disadvantage of being very intrusive, altering the client process’s address space. A malware can detect the presence of the monitoring solution and remove the hooks.

### 5.3. Server Monitoring: RPC Proxy

Out-of-process COM communication is accomplished through the use of RPC. The marshaled objects flow through an ALPC port from the client to the server.

Solutions to install RPC proxies are known. In some versions like [30], the proxy may be installed by changing the original RPC communication channel names stored in the object manager and setting up the proxy channel to have the original name. Each time an RPC port would be opened, the proxy will be opened instead. Installing the proxy requires application hooking on the server or client level with the prior being more complicated than the latter. A COM monitoring system may be built using RPC proxies by creating a man-in-the-middle interception sensor for the communication between the COM client and the COM server. The role of the interception sensor is to parse the marshaled data and deduce the operations to be performed and the client who requested them.

Monitoring WMI using this approach is complicated. Client calls to WMI are first sent to a *svchost.exe* process and then executed by a *wmiprvse.exe* process. Because of this, each WMI call needs to be monitored using two proxies: one for client communication with *svchost.exe* and one for communication between *svchost.exe* and *wmiprvse.exe*.

Even though RPC call interception is a viable monitoring strategy, ALPC communication protocol parsing is challenging and predisposed to bugs that may lead to the elevation of privileges since the servers are elevated. The challenges multiply when dealing with WMI.

### 5.4. Server Monitoring: Per Thread OLE Structures

A less technologically challenging server monitoring system may be built based on inspecting a structure internal to the COM runtime, tagSOleTlsData, that resides in a thread’s Thread Environment Block (TEB) used during COM call execution in the context of the COM server.

For this monitoring solution, a monitoring system identifies COM servers and monitors their actions using normal kernel-mode sensors. When a server performs an action, the system tries to read the client process identifier (PID) of the process that requested the action from the thread’s TEB. The algorithm is as follows.


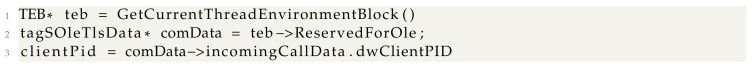


Because Kernel-mode callbacks used for file system activity monitoring may be invoked in an arbitrary context, inspecting the current thread may yield undefined behavior. This may happen if other filter layers above pend the request and resume it (or issue another one) on a system worker thread. The kernel-mode agent must obtain the requester process, switch to its address space, and then inspect its TEB. On the Windows operating systems, this can be achieved by inspecting the “Irp->Tail.Overlay.Thread” field for the monitored filesystem I/O request packet (IRP).

Monitoring WMI using this approach is again affected by the multiple levels of indirection between the original COM client and the server resolving the request (remember the *svchost.exe* process acting as an intermediary for communication between the client and the wmiprvse.exe performing the actions). The consequence of this architecture is that *wmiprvse.exe* can only be correlated to *svchost.exe*, which does not yield the original COM client.

This approach works well only for direct COM calls without an intermediary service. The approach works on all of our target Windows versions starting with Windows 7 with few changes necessary between different versions. We could not find attacks against this monitoring method that work without running code in the kernel. The main limitation is that we cannot monitor WMI with this approach.

### 5.5. Instrumentation: ETW Monitoring Window

We wanted to build a completely non-intrusive monitoring system relying only on filtering technologies such as minifilters, kernel notification callbacks, and ETW.

While researching the internals of COM, we found the interesting ETW provider *Microsoft-Windows-COM-Perf (GUID: b8d6861b-d20f-4eec-bbae-87e0dd80602b)*, which logs some information about COM object calls. Events with ID 43 (COM_ServerSyncCallStart_V2) and 44 (COM_ServerSyncCallStop) generated by this provider looked promising. One start and one stop event are generated for each COM object method call serviced by a local out-of-process COM server. These events can be correlated by matching the internal event field named *CallerTraceId*.

The time between the Start and Stop event timestamps, when matched by *CallerTraceId*, forms a *Monitoring Window* in which an out-of-process server request is being serviced.

The process ID of the client that requested the COM operation is stored in the *SourceProcessId* field of the Start event. The thread ID for the server thread that performs the actions requested by the client during the monitoring window is also provided by the aforementioned events in the *EventRecord.Header.ThreadId* fields.

**Figure 9:**
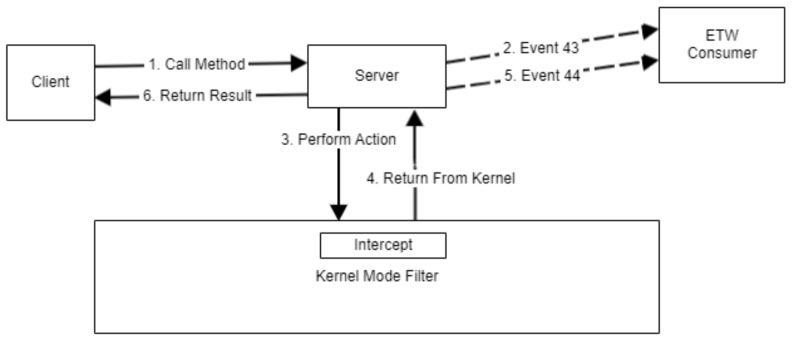
Monitoring COM using ETW and kernel mode filters.

Putting it all together, using COM-Perf, for any COM operation, we know the ID of the thread of the server that performed actions requested by the client, and we know when the operation started and when the operation finished. Using these deductions, we can build a COM monitoring system like the one presented in Figure 9. A kernel-mode sensor first identifies the COM server, and then it records the actions performed by each thread of the server together with their timestamps. Another sensor can wait for events from ETW COM-Perf and calculate the monitoring window interval, the server thread ID, and the client that requested the operation. After this calculation, the events recorded for the server thread can then be attributed to the client that requested the operation.

Monitoring COM asynchronous operations is possible through the use of *Microsoft-Windows-COM-Perf* provider events with ID 45 (COM_ServerAsyncCallStart_V1) and 46 (COM_ServerAsyncCallStop_V1).

We have implemented the ETW consumers, serializers, and deserializers required by this method and tested it on CLSID_FileOperation [41] objects. It worked well for monitoring synchronous COM calls for performing file system activity out-of-process.

Registry operations through WMI were harder to monitor because of the already mentioned fact that WMI operation leads to several other chained out-of-process COM calls. Therefore, we scaled the solution to accommodate registry operation monitoring by changing the rules for constructing the monitoring window and considering *wmiprvse.exe* as the out-of-process operation server. Under normal conditions, the solution works and correctly identifies the operation source.

Under severe stress conditions, we have noticed that *wmiprvse.exe* uses multiple threads to manage requests from clients. At the time of this study, we could not find an ETW event that gives us information that can be used to correlate wmiprvse threads with the respective clients performing the calls. This prevents us from knowing which thread services the WMI request, effectively introducing some false positives for attributing the actions to their owner processes. The false positives need to be accounted for and mitigated by the monitoring solution using this COM monitoring technique. The simple presence of such severe stress conditions may indicate malicious activity trying to blind the sensor.

The *Microsoft-Windows-COM-Perf* ETW provider on which we constructed the Monitoring Window technique is not available on Windows 7. Therefore, the solution works only for Windows 8.1 and 10.

On Windows 10, Microsoft offers some WMI monitoring capabilities out-of-the-box through events from the *Microsoft-Windows-WMI-Activity* (GUID: *1418ef04-b0b4-4623-bf7e-d74ab47bbdaa*) ETW provider. An event with ID 11 is generated when a WMI query is executed, an event with ID 22 when a method is executed, and an event with ID 23 when a WMI service creates a new process. The *Microsoft-Windows-WMI-Activity* provider is present on Windows 7 and 8.1, but it does not provide the same information as on Windows 10.

Using the WMI-Activity provider instead of the COM-Perf provider as a source of data for the monitoring window algorithm, the solution works correctly even under stress conditions and can identify the client who requested COM operations or WMI operations.

Please note that using the WMI-Activity provider directly, without the monitoring window algorithm, does not work for monitoring registry operations because the event with ID 22 that shows when a WMI method is executed does not log the parameters for the method.

Attacks against these ETW solutions exist but require either the execution of commands that can be intercepted by the security solution or the execution of malicious kernel code [58].

### 5.6. COM and WMI Monitoring Strategies Evaluation

In the previous paragraphs, we discussed strategies to identify the source of an action performed by a COM server by intercepting actions in the client, in the server, or by using already existing instrumentation data through ETW. A summary of the results for each method is presented in Table 1.

We consider binary call interception more viable than object proxies for client monitoring because they suffer from the same kind of defense evasion problems, and object proxies cannot monitor WMI.

We consider parsing per thread OLE structures to be a viable way to determine the client requesting an operation for server monitoring. We avoid using RPC proxies because the architecture is too complicated and prone to problems, and it has some hard limitations regarding WMI monitoring.

Based on ETW instrumentation, we conclude that building a monitoring window using ETW Com-Perf for generic COM objects and ETW WMI-Activity-Provider for WMI is a good solution that requires no altering to the client address space and is hard to bypass.

## 6. Further Complications: ALPC as an Out-of-Process Execution Method

ALPC is integral to to various functionalities within Windows; for instance, applications utilizing Remote Procedure Call (RPC) indirectly engage ALPC when opting for local-RPC through *ncalrpc*. Windows contains multiple security relevant RPC interfaces that allow client processes to request action such as creation of users, manipulation of services and tasks or removal of event logs.

The kernel component of ALPC exports an executive object called Port Object in order to maintain the state needed for communication. The following types of ports are present in the connection model:

- Server Connection Port —This is a named port that serves as the point where connection requests are made. It is utilized by clients to establish connections with the server, and it also receives messages sent by clients.
- Server Communication Port—This unnamed port allows a server to communicate with a specific client.
- Client Communication Port—Similarly, this unnamed port is used by a client to facilitate communication with a specific server.

**Figure 10:**
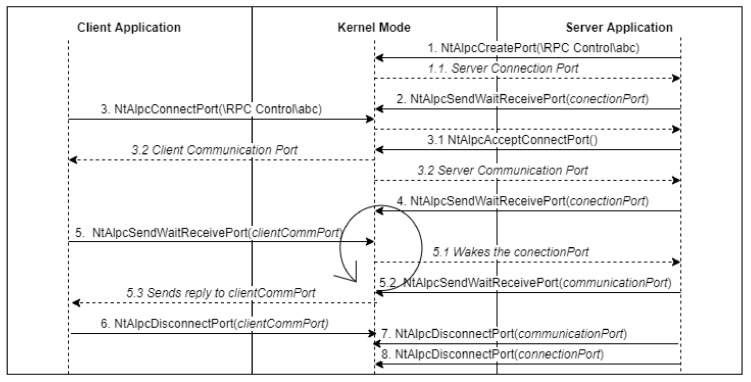
ALPC flow.

The ALPC connection model resembles the socket model. However, the APIs for creating the port, connecting to it, and transmitting messages through it remain undocumented. To have a basic client–server functionality, the setup consists of the following steps and is illustrated in Figure 10.

1. Initially, a server uses *NtAlpcCreatePort* to set up the Server Connection Port.
2. A client then tries to establish a connection using the *NtAlpcConnectPort*.
3. If the server is ready to listen, it receives a connection request message. The server has the option to accept this connection using the *NtAlpcAcceptConnectPort*. Upon acceptance, the Server Communication Port and Client Communication Port are created on the server and client sides, respectively. The server can also assign a port context to identify a client, which is a custom data structure created by the server.
4. After the connection is established, both the server and client can exchange messages using the *NtAlpcSendWaitReceivePort*.
5. Finally, once communication is complete and the ports are no longer required, either the client or the server can terminate the connection by invoking the *NtAlpcDisconnectPort*.

Monitoring ALPC calls can be accomplished via API interception inside the client or the server process. Interesting APIs are the NtAlpcCreatePort, NtAlpcConnectPort and NtAlpcSendWaitReceivePort. Other approaches such as employing creating a monitoring window based on ETW or using ALPC-related fields from the TEB may be possible and remain as future work.

## 7. Process Monitoring System Architecture

In the previous sections, we stated the out-of-process execution mechanisms like COM, WMI and ALPC for malicious behavior detection and studied several COM and WMI monitoring strategies. This section showcases the architecture of our complete dynamic detection security solution that is resistant to COM and WMI action source spoofing attacks. We built the solution starting from some specifications presented in [34] and adding a COM, WMI and ALPC monitoring layer on top of those specifications.

**Figure 11:**
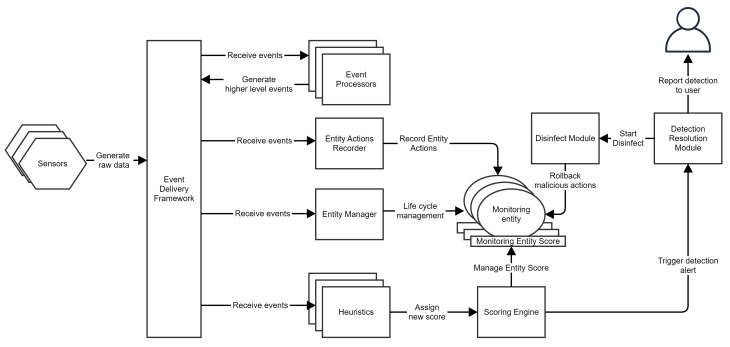
Monitoring system architecture.

The architecture of our system is presented in Figure 11. The solution is built on a data acquisition layer formed by multiple sensors sending data over an event delivery framework to which multiple layers of event processors are connected.

As sensors in kernel mode, our solution uses a minifilter driver that registers callback routines. The solution is notified whenever changes occur in the file system, registry keys, or when processes are created. At the User Mode level, the actions performed by processes are filtered by sensors created using API interception (hooking) through a DLL injected [59] into the monitored process.

The event processors interpret sensor data and generate some higher-level events that describe application behavior.

An entity manager receives events such as process creation, code injections, or service starts and maintains relations between processes using a slightly improved algorithm for group management than the one in [34]. We add processes to the same monitoring entity if they are linked by a process–child relation, a code injection relation or if a process indirectly starts another process using Windows tasks or services.

All data on the event delivery framework are available to a series of special event processors called heuristics. A heuristic is a set of one or more algorithms that interpret behavior described as events and assign a score that measures the probability that action is malicious. If the measured probability is non-zero, the heuristic assigns some points to the monitoring entity that performed the action. Point management is accomplished by a Scoring Engine.

A detection is triggered if a monitoring entity score reaches a certain threshold. This detection information is forwarded to a Detection Resolution Module responsible for notifying the user about the detection and starting the disinfection process.

For disinfection, our system uses an entity actions recorder, which receives behavioral information events, extracts information required for disinfection from them, and stores this information in a context associated with a monitoring entity. For example, when a process changes a sensible registry key, the old and new value data are stored as data associated with the monitoring entities the process belongs to.

When the Detection Resolution Module receives a detection alert, it calls the Disinfect Module. This module simply reads data stored by the entity actions recorder and rolls back any changes to the system.

### 7.1. Adding COM, WMI and ALPC Monitoring to System

**Figure 12:**
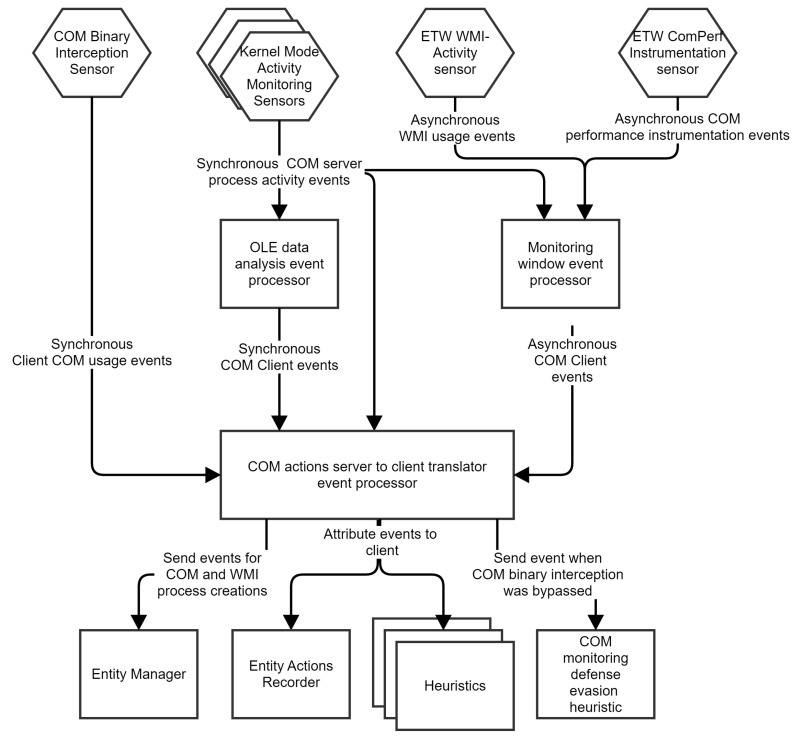
COM and WMI monitoring architecture.

To correctly identify the source of actions performed using COM and WMI, we augmented the monitoring system using several sensors, event processors, and even some heuristics. We also added several new rules for entity management. A summary of the changes is presented in Figure 12.

For a COM monitoring strategy, we decided to use a combination of some of the methods described in Section 5. Using multiple strategies makes it possible to build a more robust system that provides a defense-in-depth mechanism against malware that tries to disable the sensors.

The first and most basic sensor is the COM Binary Interception Sensor. This sensor generates synchronous events whenever a client calls a method from a COM interface that is of interest to the system. As previously stated, we know that a malicious client may remove the hooks placed by the sensor, so we detect the removal of hooks by correlating data extracted by this sensor with data extracted by the other sensors.

The second layer of COM activity monitoring is implemented in an OLE data analysis event processor. This processor inspects per-thread OLE structures and attributes events performed by the server to clients.

The third level of COM activity monitoring is implemented using ETW. We implement the monitoring window detection approach using a COMPerf sensor and a WMI-Activity sensor that sends data to a monitoring window event processor.

All COM monitoring logic eventually generates events processed by a “COM actions server to client translator” event processor that identifies the client that requested the actions performed by the server. This event processor has two main responsibilities: use a simple synchronous COM Binary Interception Sensor to assign actions performed by servers to clients and detect when this sensor was compromised by checking if the harder to defeat communicating monitoring strategies report the same data.

A special heuristic triggers a detection when the system detects that the binary interception sensor was compromised.

If the sensor is not compromised, all events sent for a COM server process are also sent for a COM client process. This makes the behavior monitoring system immune to spoofing and able to detect malware that uses COM and WMI as though all actions were executed in-process.

Please note that our system does not need binary interception on Windows 10 and above to monitor COM. Instead, the “COM actions server to client translator” event processor can work solely by using the ETW monitoring window algorithm and the parsing of OLE data. We consider this a great achievement. We kept using hooking as part of our defense-in-depth architecture because the removal of the hooks is a strong indicator that a monitoring entity is an advanced malware.

For ALPC monitoring, we implemented a simple hook-based approach where we monitor the *NtAlpcCreatePort*, *NtAlpcConnectPort* and *NtAlpcSendWaitReceivePort*. Whenever a *NtAlpcCreatePort* or *NtAlpcConnectPort* event happens, we verify if the port name is under monitoring. If it’s not monitored, we ignore the event; otherwise, we record the port handle. When *NtAlpcSendWaitReceivePort* is detected, we check if the port handle is recognized and if the RPC interface is relevant. If neither condition is met, we disregard the event. This approach offers the potential of a real-time blocking capability.

### 7.2. Main Improvements to Detection Introduced by COM, WMI and ALPC Monitoring

Adding COM monitoring to the security solution improves detection and remediation on multiple fronts.

First, by monitoring process creation through WMI Win32_Process class and by also monitoring other ways to spawn process via COM like manipulating services with objects such as *ITaskService* [60], or other various task scheduler scripting objects [61], we can improve the construction of monitoring entities. Whenever an indirect process creation is detected, we add the child process to the same monitoring entity as the parent.

Second, by correctly identifying the source of process creation, Registry, or file system modifications, heuristics have access to more of the behavior exhibited by a monitoring sample. This improves detection and may make it so malware is detected earlier before it has a chance to perform more destructive actions.

Third, knowing the correct source of action improves remediation post detection. The Disinfect Module can roll back changes to the system even if they were made through COM.

Finally, some malicious actions can only be performed using COM and WMI; thus, they may only be observed using COM monitoring. An example of such actions may be the use of *IFileOperation* for building User Account Control (UAC) [62], bypass attacks [63], or the set up of persistence using WMI.

## 8. Tests and Results

### 8.1. Malware Detection Results

Our detection system and our proposed methods work on all newer versions of Windows 10 and 11 out of the box without needing any changes to the configuration of the OS. The solution without the ETW Monitoring Window was tested for all Windows 7 and above versions of the OS.

We tested our detection system against large datasets of malware and clean processes and measured the spread of evasive malware that use COM and WMI for defense evasion. The dataset is private and was collected between 2018 and 2024 from various internet locations, honeypots, and voluntary sample submissions. We used methods similar to those described in [64] to make sure no label in the malware dataset exceeds 0.5% of the entire dataset. For each malicious sample, we had access to execution traces from our security solution for Windows 10 and 11.

As far as we know, at the moment of writing this paper, the larger datasets available to researchers do not contain malware that can be detonated inside virtual machines.

We analyzed two types of malware distribution methods, the classic method of delivery of an infected executable file as well as the more modern fileless method of delivery that relies on living-of-the-land attacks, i.e., using executable files already present on the machine (referred to as lolbins) to run the malicious code. The second category contains, among others, malicious scripts or command lines interpreted by scripting engines such as *Powershell* or *wscript*, DLLs executed using *rundll32*, or malicious documents with macros or exploits interpreted by components of the Microsoft Office suite.

For analysis of the spread of COM in malware, we looked at 402,000 malicious hashes and 681,000 lolbin executions detected by the security solution. In addition, we also measured the use of COM for non-malicious uses by looking at 2,451,000 clean executable files and 2,445,000 lolbin executions. A summary of the test dataset is presented in Table 2.

The first interesting observation derived from data is that the percent of samples that use COM and WMI in malware and clean applications from the dataset is very similar. For normal executable, 3.7315% of the infected samples and 7.1775% of the clean samples use COM in some way. For executions of lolbins via scripts, command lines, or other interpreted input data, 5.6129 % of the malicious executions observed and 5.8347% of the clean executions used COM.

The second observation is that COM and WMI usage, in general, is not an indicator of maliciousness. Treating it as such predisposes the system to many false positives. The context in which COM and WMI are used is very important for detection.

The third relevant observation is that ALPC is far more used than COM and WMI, and the same for COM and WMI, the usage of ALPC is normal for Windows applications; most of the time, system library functions use ALPC without the developer seeing it directly.

For a more in-depth look at how the out-of-process execution nature of COM, WMI and ALPC is exploited, we counted the number of samples that use some security-relevant out-of-process COM and WMI objects and RPC interfaces. A summary of the results is presented in Table 3.

For COM file system activity, we found eight lolbin executions and two malicious samples that used COM for evasion. In our dataset, we have also observed 120 clean executables and 384 lolbin executions using IFileOperation. Furthermore, as a special case of file system activity, we counted 96 malicious executables that use IFileOperation for a UAC bypass and, as expected, 0 clean executions that do this.

As an extra note, we looked at two more classes of out-of-process COM objects we considered important. First, we looked at Task and Service manipulation via COM and found it very present in both clean and malicious executions. Overall, 647 malicious lobins and 1100 malicious executables interacted with the tasks or services interface. Many clean executions of lolbins (90,547) and clean executables (18,843) also interacted with tasks and services using COM. Second, we looked at how samples interact with the Volume Shadow Service (VSS) involved in backing up user data. We found that 164 loblbin executions and 9 malicious executables interacted with VSS via COM to disable backups. We have also seen 2566 lolbin executions and 695 clean executables interacting with VSS.

For WMI activity, we looked at WMI queries, persistence, process operations, and registry operations.

WMI queries are present in many malicious executions, 2351 lolbin executions, and 9235 malicious executables as well as a large number of clean executions, 7686 lolbin executions, and 3022 clean samples.

WMI persistence is seldom used; we have seen 131 malicious lolbin executions, 6 malware, 124 clean lolbin executions, and 33 clean executables that register event consumers.

Process creation and registry operations using WMI were used only by malware in our dataset. Overall, 2112 malicious lolbin executions and 540 malware launched processes using WMI WIN32_Process. In addition, 846 malicious lolbin executions and 99 malware set dangerous registry keys using WMI.

Our dataset did not contain samples that attempted to tamper with the COM binary interception hooks.

For RPC interfaces used over ALPC, we looked at three relevant interfaces: the samr interface (12345778-1234-ABCD-EF00-0123456789AC) used for user and group manipulation on the machine, the lsarpc interface (F6BEAFF7-1E19-4FBB-9F8F-B89E2018337C) used for security policy management and the IEventService interface (F6BEAFF7-1E19-4FBB-9F8F-B89E2018337C) that can be used to manage the windows event logs. For all three interfaces, we see both clean as well as malicious executions where the interface is used in similar percentages. This again confirms that the security solution probably sees ALPC used from libraries of the operating system as part of some other operations.

### 8.2. Performance Impact Evaluation

In order to test the performance impact of the monitoring system, we first used micro-benchmarks that measure individual operation overheads, similar to the ones described by AV-Test, which is a trusted anti-malware solution testing laboratory [65]. The considered use cases for testing were file copy on the same machine and over a network, video and photo encoding, installing and running popular applications, and creating and unpacking archives.

The test was performed on a system with an Intel Core i5-4460 @ 3.20 GHz processor, 16 GB DDR3 RAM, and a Samsung 850 EVO SSD.

**Figure 13:**
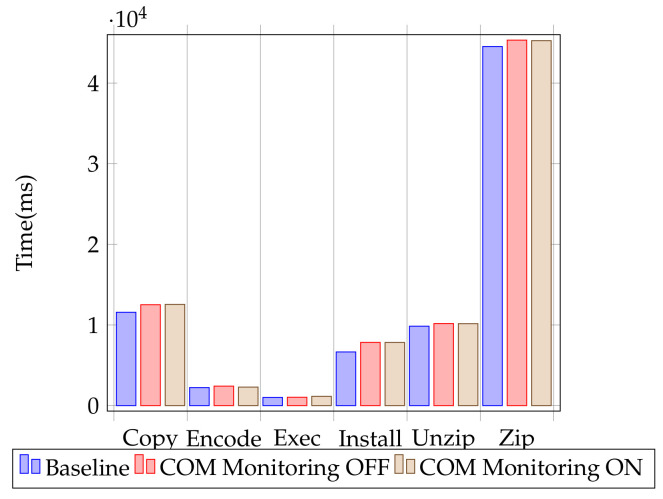
Performance test results for user activity.

Test consistency was assured by disabling Windows Defender, Windows Updates, and backup tasks. Each test was run a total of 12 times. The best and worst results were eliminated, and an average of the remaining 10 test results was computed. The error margin for the test is +/−0.15%. The averages are displayed in Table 4 and Figure 13. Comparing the results, we see that the differences between the solution with COM monitoring off and with COM monitoring on are within the test error margin.

**Figure 14:**
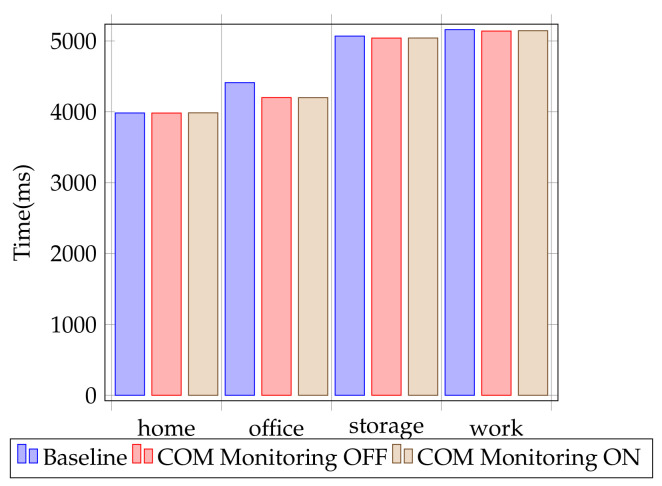
Performance test results PCMark 10.

We measured the overhead introduced by COM monitoring for end-users for real-life applications by running the PCMark 10 benchmark [9]. PCMark has the benefit of using the Microsoft Office suite (which uses COM extensively) for one suite of performance tests. Results are shown in Table 5 and Figure 14. The scores for the solution with COM monitoring off and with COM monitoring on are very similar.

## 9. Discussion

In our paper, we showed how COM, WMI, and ALPC can be used by malware for defense evasion to spoof the source of process actions that affect the system. The sensors of security solutions will first see legitimate Windows processes perform these actions and need to use specific techniques to reverse the spoofing and identify the actual source of the action. This is specifically relevant to an SOC because if the spoofing is successful, analysts may see Windows processes performing dangerous actions and ignore them.

Our tests show that our proposed solution, which combines multiple detection strategies, works for malware detection. We did not test commercial or free security solutions, as this is a different topic of research that does not align well with the author’s affiliations. However, in our paper, we show how to build programs that use COM, WMI, and RPC over ALPC as means for defense evasion. This may help other security researchers build test applications to check if their security solution is effective for monitoring COM, WMI, and ALPC usage. We suggest researchers first start such tests with Koadic [9]. If their security solution detects it dynamically (i.e., it sees behavior correctly), the researchers should consider what we show in Table 1 and test their solution for weaknesses.

We used a large private collection of malware to test the effectiveness of our security solution. The tests should be taken as proof that real-world malware uses COM, WMI, and ALPC directly. Our tests aim to show some of the more critical COM, WMI, and ALPC-related actions that should be detected by a security solution and balance this with results from testing clean samples so that we can show that COM, WMI, and ALPC usage in itself is not a sign of maliciousness.

Our performance tests show that security solutions can monitor COM, WMI, and ALPC without adding significant performance impact. This should encourage security solution developers to consider adding such monitoring capabilities to their products.

Our paper did not touch on the topics of protection methods against COM, WMI, and ALPC-based malware, so we mention some ideas that may help readers here.

First, many dangerous COM objects require the user to be an Administrator on the machine. Using low-privileged users for day-to-day activities drastically reduces the attack surface regarding COM. Readers should note that due to how interfaces like the previously mentioned *IFileOperation* work, restricted tokens for administrator users are almost equivalent to non-restricted tokens because UAC can be bypassed with them. As an added benefit, low-privileged users are also severely restricted in terms of which RPC interfaces they can use.

Second, security analysts and system administrators should consider disabling COM objects in Office [66]. Our work shows that many malicious documents use COM.

Third, WMI can be disabled on any machine by stopping the winmgmt service [67]. This may cause some applications to malfunction and may not be suited to all environments.

Finally, Distributed COM can be disabled if it is not used in the organization of the SOC. Detailed information about how to disable DCOM is provided by Microsoft [68]. Remote WMI and DCOM are closely related. Microsoft provides a lot of information about how remote WMI works and how it can be secured in [69].

## 10. Conclusions

In our paper, we set out to address the challenge of detecting and mitigating malware that uses COM, WMI, and ALPC for defense evasion. Our primary objective was to design and evaluate a robust monitoring system that provides a defense-in-depth mechanism against such evasive techniques.

We began by detailing how COM works internally and how it can be leveraged by malicious actors to spoof behavioral malware detection systems. We then proposed several solutions for monitoring COM and WMI activity, critically discussing their advantages and disadvantages. Our approach combines classic monitoring techniques with two novel methods we introduced, culminating in a comprehensive defense-in-depth architecture.

Additionally, we extended our analysis to ALPC, demonstrating its potential for out-of-process execution and its relevance in the context of malware detection.

We integrated these monitoring capabilities into a dynamic detection system that not only detects evasive malware with high accuracy but also offers remediation capabilities with minimal performance impact. Our extensive testing, using a large dataset of telemetry data, confirmed that a significant number of malicious programs exploit COM and WMI for defense evasion.

Our findings underscore the importance of including COM, WMI, and ALPC monitoring in security solutions. We hope that our work will guide researchers and security professionals in developing robust defenses against sophisticated spoofing attacks that leverage these technologies. 

## Figures and Tables

**Table 1:** Comparison of monitoring strategies.

Solution	Level of Intrusion	Can Monitor COM	Can Monitor WMI	Known Attacks without Elevated Privileges	Implementation Difficulty
Client: object proxies	Medium -requires set up of proxy	YES	YES	YES -load original library	Easy -Technology is built into COM
Client: binary call interception	High -requires hooks in client	YES	YES	YES -remove hook	Medium -Requires set up of hooks
Server: RPC proxy	High -requires hooks in server	YES	YES -but with limitations	NO	Very hard -Requires protocol parsing
Server: Per thread OLE structures	None	YES	NO	NO	Easy -Requires reading of data from TEB
Instrumentation: COM-Perf	None	YES	YES -but with limitations	NO	Medium -Monitoring window algorithm is difficult to implement
Instrumentation: WMI-Activity-Provider	None	NO	YES	NO	Medium -Requires the same monitoring window algorithm as COM-Perf

**Table 2:** Testing dataset summary.

	Samples	Use COM	Use COM (%)	Use ALPC	Use ALPC (%)
**Infected lolbin executions**	681,000	38,224	5.6129	104,154	15.2942
**Malware**	402,000	15,001	3.7315	53,867	13.3997
**Clean lolbin executions**	2,445,000	142,659	5.8347	337,416	13.8002
**Clean**	2,451,000	175,921	7.1775	350,983	14.3199

**Table 3:** Out-of-process COM usage summary.

Type of Activity	Infected Lolbin Execution		Malware		Clean Lolbin Executions		Clean	
	**Number**	**%**	**Number**	**%**	**Number**	**%**	**Number**	**%**
**COM Filesystem Activity**	8	0.0011	2	0.0004	384	0.0157	120	0.00489
**COM UAC Bypass Using Auto-Elevation**	0	0	96	0.0238	0	0	0	0
**COM Tasks and Services**	647	0.0950	1100	0.2736	90,547	3.7033	18,843	0.7687
**COM Volume Shadows Manipulation**	164	0.0240	9	0.0022	2566	0.1049	695	0.0283
**WMI Query Info**	2351	0.3452	9235	2.2972	7686	0.3143	3022	0.1232
**WMI Register Persistence**	131	0.0192	6	0.0014	124	0.0050	33	0.0013
**WMI Process Launch**	2112	0.3101	540	0.1343	0	0	0	0
**WMI Registry Activity**	846	0.1242	99	0.0246	0	0	0	0
**ALPC samr interface**	412	0.06	312	0.0776	451	0.018446	621	0.0184
**ALPC lsarpc interface**	5142	0.755	4852	1.2068	56,194	2.2298	31,954	2.29832
**ALPC IEventService interface**	42	0.0061	82	0.0203	234	0.0095	125	0.0051

**Table 4:** Test results for user activity (lower scores are better).

	Baseline	Solution without COM Monitoring	Solution with COM Monitoring	Impact Added by COM Monitoring
copy files	11,576.53	12,512.88	12,552.2	**−0.0031%**
encode	2236.29	2417.02	2297.45	**+0.0494%**
execute	1015.19	1035.6	1154.49	**−0.1148%**
install	6655.35	7840.25	7834.045	**+0.0007%**
unzip	9851.94	10,179.72	10,164.71	**+0.0014%**
zip	44,542.71	45,332.42	45,267.48	**+0.0014%**

**Table 5:** Results for running PCMark (larger scores are better).

	Baseline	Solution without COM Monitoring	Solution with COM Monitoring
**home score**	3984	3983	3986
**office score**	4412	4201	4200
**storage score**	5069	5041	5042
**work score**	5160	5140	5145

## Data Availability

The authors do not have permission to disclose the data.
